# Books and Pamphlets Received

**Published:** 1885-06-20

**Authors:** 


### BOOKS AND PAMPHLETS RECEIVED.

A PRACTICAL TREATISE ON THE DISEASES OF THE EAR, including a sketch of Aural Anatomy and Physiology by D. B. St. John Roosa, M.D., LL.D., Prof. of Diseases of the Eye and Ear in the New York Post Graduate Medical School and President of the Faculty ; Surgeon to the Manhattan Eye and Ear Hospital ; Consulting Surgeon to the Brooklyn Eye and Ear Hospital ; formerly Professor of Opthalmology in the University of the City of New York and of Diseases of Eye and Ear in the University of Vermont, etc., etc. Sixth Edition revised and enlarged. New York : William Wood & Co.

We have in the above a revised and enlarged edition of one of the best and most approved works on the Diseases of the Ear now in existence. During the eleven years since the first edition was issued it has maintained its popularity, and the present edition is evidence of the increasing demand for the work.

It contains 700 octavo pages and is elegantly and neatly printed in large, beautiful type, and illustrated with numerous cuts.

It is unnecessary to detail the plan, arrangement and matter of a work of such acknowledged merits and reputation. It should be in the library of every specialist on the Ear, and of every progressive medical student and practitioner of medicine.

SCARLET FEVER, and Certain Suggestions for its Treatment in accordance with the most recent advances in science and experience by T. Griswold Comstock, M.A., M.D., St. Louis, Mo.

GENITAL REFLEXES, the result of an Abnormal Physical Condition of the Genital Organs known as Phimosis by T. Griswold Comstock, M.A., M.D., St. Louis, Mo.

TRACTS ON MASSAGE. No. 1, the Art of Massage, translated from the German of Reibmayr, with notes, by Benjamin Lee, A.M., M.D., PH.D.

TYPHOID FEVER AND LOW WATER IN WELLS, by Henry B. Baker, M.D., Lansing, Mich.

